# A comparison of intima media thickness in the common carotid artery, the bulb and plaque area as predictions of incident atherosclerotic events

**DOI:** 10.1371/journal.pone.0294722

**Published:** 2023-11-20

**Authors:** Lars Lind

**Affiliations:** Department of Medical Sciences, Uppsala University, Uppsala, Sweden; Keio University School of Medicine, JAPAN

## Abstract

**Background and aims:**

There is a debate on how to evaluate carotid artery intima-media thickness (IMT). We here compared IMT of the common carotid artery (CCA) and bulb with plaque area regarding incident atherosclerotic disease.

**Methods:**

In the PIVUS study (age 70 at baseline, 53% women, n = 856), IMT-CCA, IMT-bulb and plaque area were measured at ages 70, 75 and 80 years and these three measurements were used in updated Cox proportional hazard analysis.

**Results:**

Over 15 years follow-up, 135 individuals experienced a first-time atherosclerotic disease (myocardial infarction or ischemic stroke). IMT-CCA was not significantly related to this composite endpoint (*p* = 0.10). IMT-bulb was significantly related to the endpoint (*p* = 0.003), but this relationship was attenuated following adjustment for CVD risk factors (*p* = 0.02). On the contrary, plaque area was consistently related to incident atherosclerotic disease also following adjustment for CVD risk factors (*p*<0.001). When added on top of traditional risk factors, both IMT-bulb and plaque area, but not IMT-CCA, improved the discrimination compared to the traditional risk factors (+5.2%, *p* = 0.0026 for IMT-bulb, +3.8%, *p* = 0.013 for plaque area and 0.0% for IMT-CCA).

**Conclusion:**

In elderly subjects, both IMT-bulb and plaque area improved the discrimination regarding incident atherosclerotic disease when added to traditional risk factors. This was not seen for IMT-CCA. IMT-CCA was therefore inferior compared to the other two carotid artery ultrasonographic measurements in this sample of elderly subjects.

## Introduction

Ever since the introduction of carotid artery ultrasound in research of atherosclerosis, carotid artery intima-media thickness (IMT) has been evaluated. Since the ultrasound used in the clinic or in most epidemiological studies do not have the potential to differentiate the intima from the media layer, those two components of the vascular wall are lumped together into one measurement. IMT could be measured at multiple sites, near and far wall, in the common carotid artery (CCA), the bulb or the internal carotid branch, and with different angles. Furthermore, since atherosclerosis mainly originates in the bulb/proximal internal artery, it is possible to measure IMT-CCA without an involvement of a potential plaque or by including the plaque in the measurement. In addition, some investigators use the mean value of IMT at several sites, while many large population-based studies have only evaluated IMT in the far wall of CCA. Even so, the mean value of several measurements over a 10 cm distance could be used or the maximal value over this distance. Thus, the literature on IMT is based on a variety of definitions of IMT, as reviewed by Naqvi and Lee [1]. Despite this diversity in determinations, it has been shown in meta-analyses that IMT is related to both myocardial infarction and stroke, the two major atherosclerotic diseases [2–4]. However, the improvement in risk prediction by adding IMT to traditional risk factors has not been that great, so measurement of IMT for risk stratification is not included in major guidelines on this topic **[5–7].** In recent years it has become apparent that IMT-CCA and IMT-bulb should be regarded as different entities, although being correlated. First, IMT-CCA was previously found to mainly be related to hypertension, while IMT-bulb was mainly related to high LDL-cholesterol and smoking [8]. Secondly, several of the genes and a polygenetic score for coronary heart disease are related to IMT-bulb, but not to IMT-CCA [9, 10]. Third, IMT-bulb and IMT-CCA are partly related to different plasma protein profiles [11]. Fourth, IMT-CCA is mainly related to stroke, while IMT-bulb is mainly related to coronary heart disease [8]. Thus, there are several reasons to believe that IMT-CCA and IMT-bulb are governed by different processes and therefore should not be treated as equal measurements.

Just like measurements of IMT, multiple definitions on plaque in the carotid artery has been used. Using a cut-off at 1.2 or 1.5 mm for IMT-bulb is a commonly used definition, while others have demanded a local thickening of IMT by 50% or some other millimeter-based cut-off [1]. Some studies have only used plaque as a binary variable, while others have measured the total plaque area in both carotid arteries. Despite this discrepancy in definitions, a meta-analysis of 11 studies has shown that plaque is related to myocardial infarction, in a stronger fashion than IMT [12]. The majority of studies in that meta-analysis used carotid plaque as a binary variable, and it might well be that a more fine-tuned variable describing plaque, such as plaque area, might be an even better predictor than a binary plaque variable.

The present study was undertaken to compare different continuous measures of carotid atherosclerosis in an elderly population (IMT-CCA, IMT-bulb and plaque area). In order to compare the predictive power of IMT-CCA, IMT-bulb and plaque area, these three carotid artery characteristics were measured at three occasions in the population-based PIVUS study [13] over a 10-year period and were related to incident atherosclerotic disease (myocardial infarction or ischemic stroke) during a follow-up over 15 years. Although several studies have compared IMT and plaque in terms of risk prediction, the novel features in the present study is to compare three different continuous measures of carotid atherosclerosis in an elderly population using repeated measurements. The hypothesis tested was that plaque area would be superior in prediction of incident atherosclerotic disease compared to IMT measurements. As a secondary aim, the association between baseline IMT-CCA or IMT-bulb and plaque area 5 years later in those without plaque at baseline was investigated.

## Material and methods

### Sample

The Prospective Investigation of the Vasculature in Uppsala Seniors (PIVUS) study started a random recruitment of men and women aged 70 years living in Uppsala City (Sweden) in 2001. Three years later 1,016 individuals (50% women) had been included. The participants were offered reinvestigations at age 75 and at age 80. 823 subjects attended the reinvestigation at age 75, and 604 at age 80.

At the age of 70 years, 73 individuals had a history of myocardial infarction and 39 had a history of stroke (8 had both diagnoses). These subjects were removed from further analyses.

The study was approved by the Ethics Committee at Uppsala University and followed the Declaration of Helsinki ethical guidelines. Each participant gave their informed written consent.

### Risk factor evaluation

All subjects were investigated between 8 and 11am after an overnight fast. Glucose and lipids were measured by standard techniques. Blood pressure was measured in the supine position after 15 min rest by a mercury sphygmomanometer. Height and weight were recorded, and body mass index (BMI) calculated. Smoking history was obtained by a questionnaire. Diabetes was defined as either a history of diabetes or a fasting glucose ≥7.0 mmol/l.

### Carotid artery ultrasound

**Fig 1:**
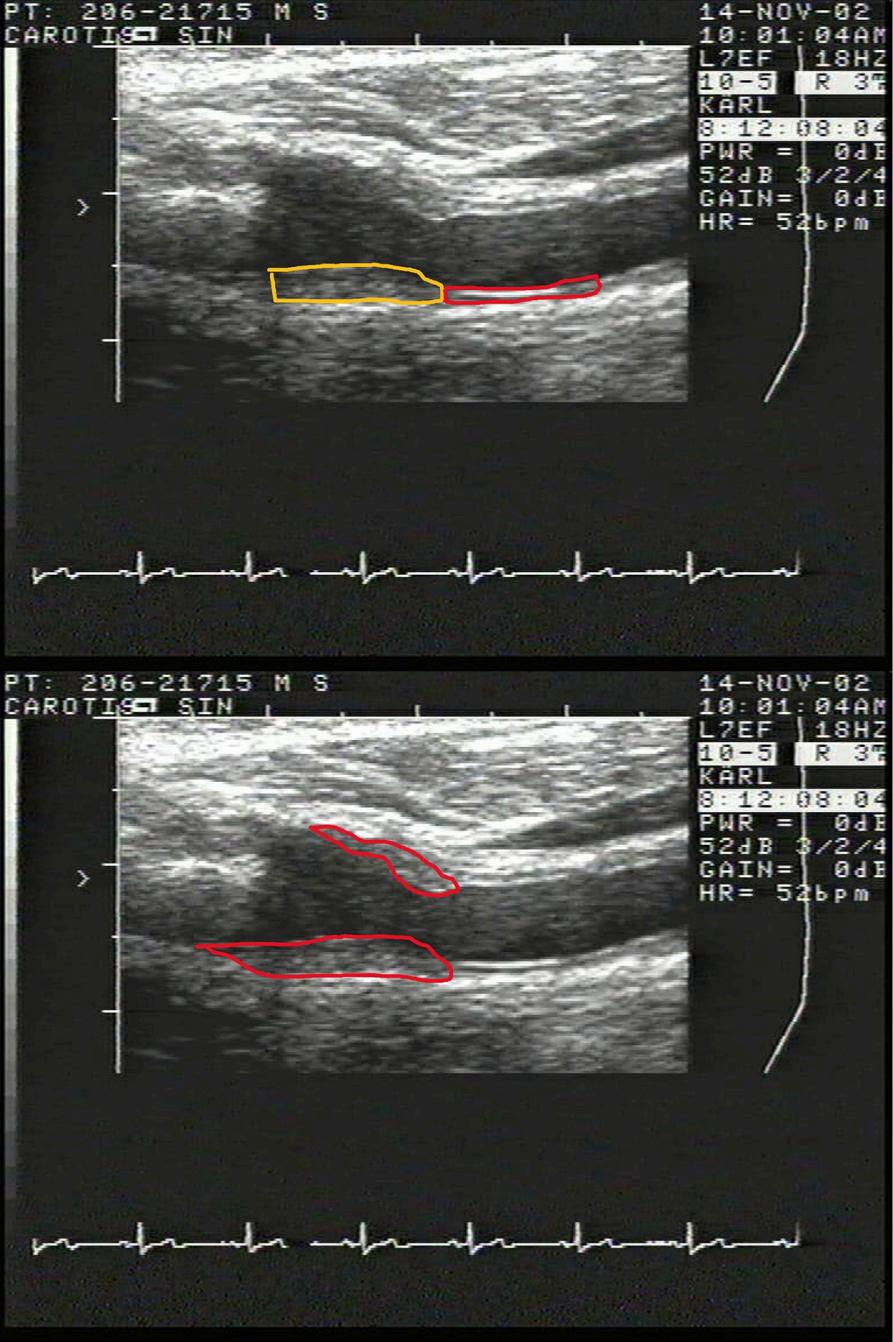
Two images of an individual with plaque. In the upper panel, the part of the common carotid artery (CCA) that is used for measurement of the intima-media thickness (IMT) is shown in red, while the part that was used for measurement of IMT in the bulb is shown in yellow. This measurement is performed in a semi-automated fashion. In the lower panel, a region of interest (ROI) in red is placed both around the plaque part being visualized in the far wall and in the part seen in the near wall. This was done by hand.

Both carotid arteries were scanned by a 10 MHz ultrasonographic probe (Accuson XP, Mountain View, CA; USA). IMT was measured in the far wall of CCA during a 10 mm segment proximal of the bifurcation. Far wall IMT-bulb was measured from the beginning of the bifurcation and a 5–10 mm segment was evaluated. Both IMT measurements were evaluated by a semi-automated software (AMS, Gothenburg, Sweden) which measures IMT 10 times per mm and reports the mean value of those recordings. IMT-CCA and IMT-bulb are given as the mean of the left and right arteries. Presence of a plaque was defined as a local thickening of IMT of more than 50% of the surrounding IMT. A region of interest (ROI) was manually placed around each plaque and the area was measured by the software. Plaque area is defined as the sum of the plaques in both arteries. For a detailed description of the carotid artery ultrasound procedure, please see [14] and Fig 1.

The measurements of IMT were repeated within a week in 30 young and middle-aged individuals giving a coefficient of variation of IMT-CCA of 7.2% and 9.2% for IMT-bulb.

### End-point definition

The participants were followed for 15 years regarding incident atherosclerotic events (as defined as either myocardial infarction or ischemic stroke). The Swedish cause of death register and hospital in-patient register were merged with the unique Swedish personal id-number of each participant. Both fatal and non-fatal events were included in this composite endpoint, defined by ICD-10 codes for myocardial infarction (ICD-10 code I21) or ischemic stroke (codes I63.0–5 and 7–9). The diagnoses were validated by reading the electronic medical records.

### Statistics

When subjects with a technically poor ultrasonographic examination and those with prevalent myocardial infarction or stroke at baseline (age 70) were excluded, 856 individuals remained at age 70.

For the primary aim, Cox proportional hazard analysis with updated values for both the carotid artery measurements, as well as for the traditional risk factors (systolic blood pressure, diabetes, BMI, LDL and HDL-cholesterol, smoking) will be used. Thus, each investigation (70, 75 and 80 years) will be treated as a separate baseline with 5 years follow-up. The three carotid artery measurements will be evaluated in separate models. In the first set of models, adjustment is only performed for age and sex. In the second set of models, additional adjustment for the traditional risk factors was performed. In a third set of models, adjustment for the traditional risk factors plus treatment vs hypertension or diabetes and statins use was performed.

The association between the three carotid artery measurements and incident atherosclerotic events were visualized using restricted cubic splines with three knots (10^th^, 50^th^ and 90^th^ percentile.

In addition to the Cox proportional hazard models, each of the three carotid artery measurements will be evaluated regarding discrimination using C-statistics and logistic regression models with robust standard errors. First, the C-statistics for each of the three carotid artery measurements alone was evaluated. Second, it was evaluated if addition of any of the three carotid artery measurements to the traditional risk factors could improve discrimination compared to a model with the risk factors only.

For the secondary aim, Spearman rank correlation was used to relate IMT (CCA or bulb) to plaque area at age 75 in the subjects being free of plaque at age 70.

STATA16.1 was used for the calculations (Stata inc, College Station, TX, USA).

## Results

Basic characteristics at the three examinations are given in Table 1.

**Table 1:** Mean and SD, or proportion, for studied variables in the sample when excluding subjects with prevalent myocardial infarction or ischemic stroke at baseline (age 70). IMT, intima-media thickness; CCA, common carotid artery; BMI, body mass index.

	Age 70		Age 75		Age 80	
	n	Mean (SD)	n	Mean (SD)	n	Mean (SD)
Female sex (%)	912	53	750	52	554	51
IMT-CCA (mm)	856	.89 (.16)	700	.95 (.17)	538	.95 (.15)
IMT-bulb (mm)	764	.99 (.19)	533	1.06 (.2)	441	1.24 (.36)
Plaque area (mm^2^)	856	14.0 (16.5)	700	19.8 (18.3)	538	23.8 (16.9)
Systolic blood pressure (mmHg)	908	149 (22)	749	148 (19)	552	146 (19)
HDL-cholesterol (mmol/l)	909	1.53 (.43)	749	1.51 (.46)	551	1.39 (.39)
LDL-cholesterol (mmol/l)	907	3.43 (.86)	749	3.43 (.94)	550	3.25 (.9)
Smoker (%)	911	10	744	6.2	545	3.4
BMI (kg/m^2^)	912	26.9 (4.3)	750	26.6 (4.3)	551	26.7 (4.4)
Diabetes (%)	912	11	747	13	544	16
Antihypertensive treatment	912	31	750	50	554	49
Statin treatment	912	15	750	25	554	33
Antidiabetic treatment	912	9	750	11	554	13

All three carotid ultrasound measurements increased over the 10-year period, mainly between age 70 and 75 (*p*<0.001 for all). The same pattern was seen in the subjects with measurements at all three investigations.

### Carotid ultrasound measurements and incident atherosclerotic disease

Over a follow-up of 15 years (from age 70 to 85 years), 135 subjects suffered from either a myocardial infarction or an ischemic stroke (called incident atherosclerotic disease, our primary outcome) during 15 years of follow-up (11,228 person years at risk).

As could be seen in Table 2, IMT-CCA was not significantly related to incident atherosclerotic disease, IMT-bulb was significantly related to incident atherosclerotic disease, but this relationship was attenuated following adjustment for cardiovascular disease (CVD) risk factors. Plaque area was related to incident atherosclerotic disease with a low *p*-value also following adjustment for CVD risk factors. Further addition of antihypertensive treatment, antidiabetic treatment, and statin use as confounders along with the CVD risk factors did not change the impact of the three indices of carotid atherosclerosis to any major degree compared to when adjustment for CVD risk factors were performed.

**Table 2:** Associations between intima-media thickness (IMT) in the common carotid artery (CCA), bulb and plaque area and incident atherosclerotic disease (myocardial infarction or ischemic stroke) over 15 years. The hazard ratio (HR) is given for a 1 mm change in IMT and a 1 mm^2^ change in plaque area. Three levels of adjustments are given, sex only (age same in all individuals), adjustment for cardiovascular risk factors and adjustment for cardiovascular risk factors plus treatment. All models are based on updated covariates from the investigations performed at ages 70, 75 and 80.

	Adjustment	HR	95%CI	*p*-value
IMT-CCA	Sex	2.62	0.83–8.20	0.098
	Cardiovascular risk factors	1.83	0.55–6.07	0.32
	Cardiovascular risk factors and treatment	2.02	0.61–6.65	0.24
IMT-bulb	Sex	2.42	1.34–4.36	0.003
	Cardiovascular risk factors	2.04	1.11–3.77	0.021
	Cardiovascular risk factors and treatment	2.12	1.15–3.92	0.016
Plaque area	Sex	1.02	1.009–1.025	<0.001
	Cardiovascular risk factors	1.02	1.006–1.023	<0.001
	Cardiovascular risk factors and treatment	1.01	1.005–1.022	0.001

**Fig 2:**
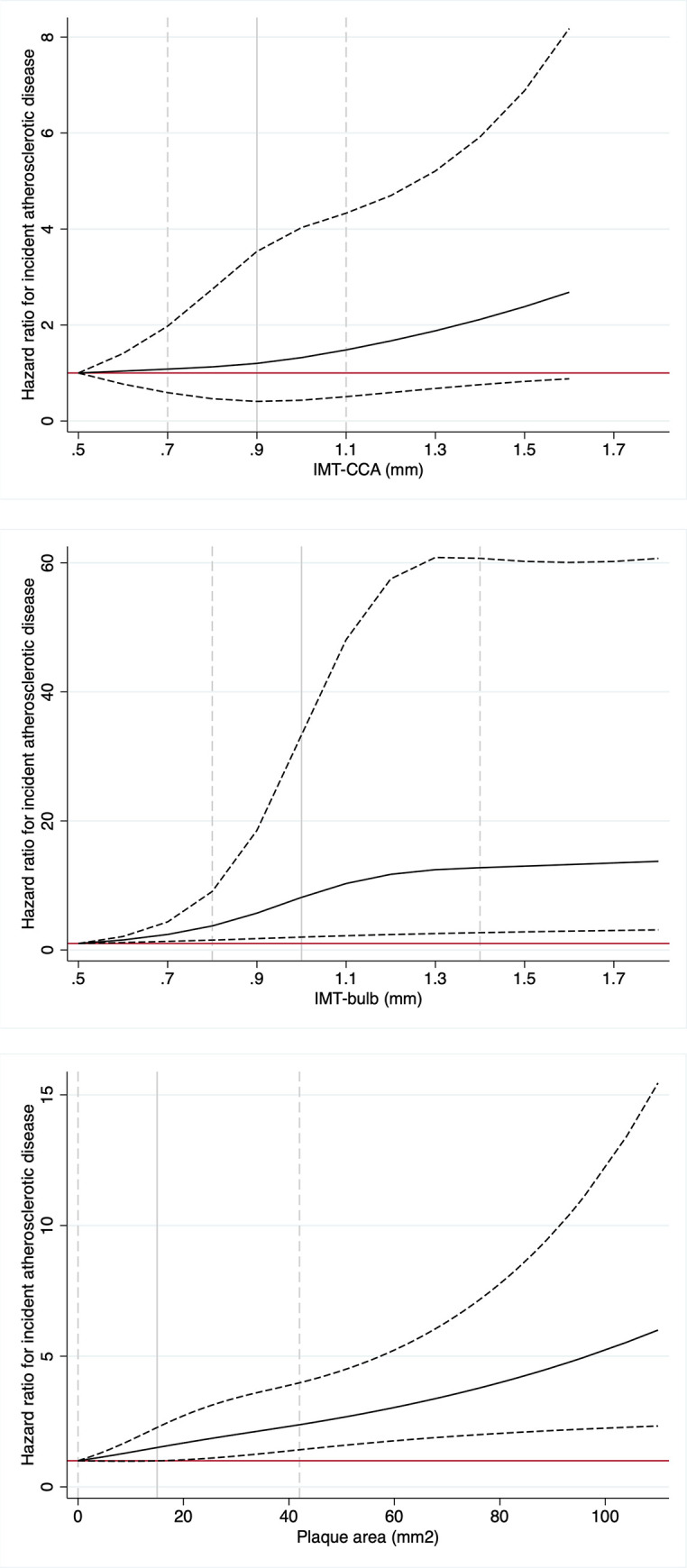
Relationships between intima-media thickness (IMT) in the (A) common carotid artery (CCA), (B) bulb and (C) plaque area and incident atherosclerotic disease (myocardial infarction or ischemic stroke) over 15 years. The lowest value for the three indices were used as reference (hazard ratio (HR) = 1) and the low and high 95%CI are given by the dotted lines. The vertical line at a HR of 1 denotes the reference. The light grey vertical lines denote the 10^th^, 50^th^ and 90^th^ percentile. Restricted cubic splines were used to model also non-linear relationships.

As could be seen in Fig 2, IMT-CCA was not significantly related to incident atherosclerotic disease at any level. For IMT-bulb the risk increase was seen already at low levels but showed a plateau after 1.3 mm. Regarding plaque area, a significant risk increase was seen when the area exceeded 25 mm^2^.

When the three ultrasonographical indices were related to incident atherosclerotic disease one by one without adjustment, the C-statistics were significantly higher for IMT-bulb and plaque area compared to IMT-CCA (0.673 for IMT-bulb, 0.650 for area and 0.553 for IMT-CCA). When added on top of traditional risk factors, both IMT-bulb and plaque area, but not IMT-CCA, improved the discrimination compared to the traditional risk factors (+5.2%, *p* = 0.0026) for IMT-bulb, +3.8%, *p* = 0.013 for plaque area and 0.0% for IMT-CCA, see Table 3 for details).

**Table 3:** Area under the curve (AUC) and C-statistics for discrimination of carotid artery ultrasound measurements and incident atherosclerotic disease (myocardial infarction or ischemic stroke) over 15 years. IMT, intima-media thickness; CCA, common carotid artery.

	AUC	95%CI	*p*-value
IMT-CCA only	0.553	0.495–0.610	CCA vs bulb = 0.0002
IMT-bulb only	0.673	0.620–0.725	Area vs bulb = 0.3018
Plaque area	0.650	0.597–0.702	Area vs CCA = 0.0060
Risk factors only	0.639	0.588–0.689	
Risk factors+IMT-CCA	0.639	0.589–0.689	vs risk factors only = 0.99
Risk factors+IMT-bulb	0.691	0.639–0.742	vs risk factors only = 0.0026
Risk factors+IMT-area	0.677	0.628–0.724	vs risk factors only = 0.013

A very similar picture emerged when NRI and IDI was used instead of C-statistics. IMT-bulb and plaque area improved discrimination and reclassification to a similar degree when added to traditional risk factors, while IMT-bulb did not improve these metrics in a significant fashion (see Table 4).

**Table 4:** Integrated discrimination improvement (IRI) and net reclassification improvement (NRI) of carotid artery ultrasound measurements when added to traditional risk factors compared to the risk factors alone regarding incident atherosclerotic disease (myocardial infarction or ischemic stroke) over 15 years. IMT, intima-media thickness; CCA, common carotid artery.

	Estimate	SE	*p*-value
NRI			
IMT-CCA	0.04	0.10	0.68
IMT-bulb	0.37	0.10	0.0003
Plaque area	0.34	0.10	0.0006
IDI			
IMT-CCA	0.0021	0.0014	0.12
IMT-bulb	0.011	0.0034	0.0010
Plaque area	0.011	0.0043	0.014

### IMT-CCA and IMT-bulb as determinants of future plaque occurrence

In the 230 subjects who showed no plaque at baseline (age 70) and had a follow-up measurement at age 80, 70% showed a plaque at age 80. The median plaque area in those subjects were 11 mm^2^ (IQR 2–22). IMT-CCA (borderline significant) and IMT-bulb at baseline were related to the change in plaque area over the 5 years (Spearman´s rho = 0.13 and *p*-value = 0.050 for IMT-CCA and rho = 0.21 and *p*-value = 0.0036 for IMT-bulb.

## Discussion

The present study showed that both plaque area and IMT-bulb were able to improve discrimination of future atherosclerotic events when added to traditional risk factors. This was not seen for IMT-CCA, despite that this commonly used measure did not actively avoid including plaques in the measurement.

### Comparison with previous literature

There is a wealth of literature showing that IMT, as well as plaque occurrence or area is related to incident atherosclerotic diseases when evaluated by Cox proportional hazard models or similar techniques [2, 3]. However, in most studies IMT have not added in predictive power when added to traditional risk factors. In those previous studies, IMT was measured in different fashions, as mentioned in the introduction, and very few studies tried to differentiate between measurements performed in CCA and the bulb, being the focus of the present investigation.

Thus, it is evident that IMT-CCA is not a good marker of subclinical atherosclerosis, as advocated by Spence [15], since it could not predict future atherosclerotic disease, was only poorly related to plaque progression in subjects initially plaque free in the present study, and does not share genetics with coronary heart disease [9, 10]. IMT-CCA should be treated as a separate entity, especially when plaques are actively not included in the measurement.

One argument that however talks for an important role of IMT-CCA is the results from a recent meta-analysis of >100 randomized clinical trials in which it was shown a significant relationship between the reduction in IMT progression over time and a reduced risk of CVDs, irrespectively of the type of intervention [16].

What about IMT-bulb? This measurement was as good as plaque area to improve discrimination of future atherosclerotic events as plaque area, does share important genes with coronary heart disease [9, 10], and was well associated with plaque progression in subjects initially plaque free in the present study. It is also measured in the bifurcation, the site where most plaques are generated. The drawback of this measurement is that it includes also the media layer and atherosclerosis mainly is a disease of the intima. This was illustrated in a study with a high-resolution 25 MHz probe by which the intima and the media could be separated and the intima, but not the media, was increased in patients with atherosclerotic disease [17]. Thus, IMT-bulb is far better than IMT-CCA to reflect atherosclerosis, but is not a pure atherosclerosis measurement and therefore should be regarded as a proxy measurement if used.

In the present study, only the thickness of IMT-bulb was measured. However, it might be that also the flow dynamics in the bifurcation could be a major player in risk assessment, since this characteristic of the carotid artery flow dynamics could be measured by ultrasound as well as by magnetic resonance imaging [18–20].

As expected, the use of plaque area was a good predictor of atherosclerotic events, in accordance with previous investigations [12]. Of interest is the evaluation performed in Fig 2 showing a significant risk increase when the area exceeded 25 mm^2.^ If this could be reproduced by others, a cut off level to be used in risk prediction could be established. 3D measurements of plaque volume might be even more appropriate than plaque area, but this has been carried out in very few large epidemiological studies.

### Strength and limitations

The novelty of the present study is that IMT was measured both in the CCA and the bulb together with determinations of plaque area at three occasions during a 10-year period in an elderly population. In this way the progression of atherosclerosis was monitored throughout the follow-up period.

This is not the largest study performed on carotid artery ultrasound, but it is evident that the sample size and number of events collected during the follow-up period was sufficient to result in highly significant associations for plaque area, the major measurement of atherosclerosis in the carotid arteries. Since IMT-CCA resulted in a 0.0% improvement in discrimination when added on top of traditional risk factors, it is not likely that a larger sample size would provide a significant result in this respect.

A limitation is the lack of reproducibility evaluation of the plaque area measurement, like for IMT-CCA and IMT-bulb. The experience is however that it is harder to define the borders of the plaque compared to the borders of the IMT, so the coefficient of variation is likely to be higher for the plaque area measurement compared to the IMT indices. Thus, it is not likely that a low coefficient of variation for plaque area would explain the better performance than IMT-CCA.

A limitation of the present study is that only elderly individuals were included, so that that the results cannot be generalized to younger individuals. In a recent study it was shown that this indeed is the case since it was found that IMT-CCA did not add predictive power in subjects >55 years of age [21]. However, carotid artery ultrasound is mainly performed in the clinic in elderly individuals, an age where the risk of atherosclerotic disease is highest, so it is of importance to obtain knowledge of IMT also in the elderly.

In conclusion, in elderly subjects, both IMT-bulb and plaque area improved the discrimination regarding incident atherosclerotic disease when added to traditional risk factors. This was not seen for IMT-CCA. IMT-CCA was therefore inferior compared to the other two carotid artery ultrasonographic measurements in this sample of elderly subjects.
